# Spatial Discrepancies Between Health and Social Security Reports as Markers of Potential Underreporting of Occupational Injuries in Brazil, 2022–2023

**DOI:** 10.1002/puh2.70400

**Published:** 2026-09-30

**Authors:** Luiza Maria Parise Morales, Samara Carolina Rodrigues, Roberta Souza Freitas, Cristiano Barreto de Miranda, Klauss K. S. Garcia

**Affiliations:** ^1^ Secretariat for Health and Environmental Surveillance Ministry of Health Brasília Federal District Brazil; ^2^ Centre for Tropical Medicine University of Brasília Brasília Federal District Brazil; ^3^ Secretariat of Science Technology and Innovation and the Economic‐Industrial Complex of Health Ministry of Health Brasília Federal District Brazil; ^4^ Sergio Arouca National School of Public Health Oswaldo Cruz Foundation Rio de Janeiro Brazil; ^5^ Department of Infectious Disease Epidemiology and International Health Faculty of Epidemiology and Population Health The London School of Hygiene and Tropical Medicine London UK

**Keywords:** epidemiology, occupational health surveillance, spatial analysis, underreporting, workplace accidents

## Abstract

**Introduction:**

Occupational injuries remain a major public health problem in Brazil, and underreporting limits the capacity of surveillance systems to support prevention and response. This study aimed to identify spatial discrepancies between health reports and social security reports of occupational injuries in Brazil.

**Methods:**

This cross‐sectional ecological study uses municipality‐level aggregated data for all 5570 Brazilian municipalities. Occupational injury records from health reports (Sinan system) and social security reports (CAT system) were analysed for 2022 and 2023. Spatial distribution was described, and spatial autocorrelation was assessed using global Moran's *I* and local indicators of spatial association. A municipality‐specific log‐ratio between health reports and social security reports was used to identify relative discrepancies between the two systems.

**Results:**

There were 745,840 injury records from the health sector and 949,466 from the social security sector. Health reports were made in 5466 (98.1%) municipalities, whereas social security reports were present in 4509 (80.9%). A total of 3998 (71.7%) municipalities recorded more health reports than social security reports, whereas 1418 (25.4%) recorded more social security reports than health reports. Low–low log‐ratio clusters, corresponding to areas where social security reports consistently exceeded health reports, were concentrated in the central‐west and southeast regions.

**Conclusion:**

The spatial comparison identified marked territorial discrepancies between health reports and social security reports in Brazil. Clusters where social security reports exceeded health reports may serve as operational markers of potential underreporting in workers’ health surveillance that may help guide geographically targeted surveillance strengthening.

## Graphical abstract

Spatial comparison of health and social security reports identified territorial discrepancies in occupational injury reporting in Brazil. Clusters where social security reports exceeded health reports may indicate priority areas for investigating potential underreporting and strengthening workers’ health surveillance.

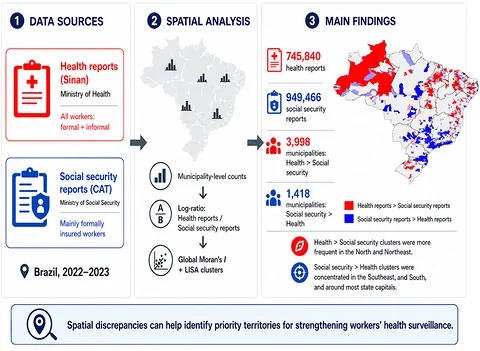

## 1 Introduction

Occupational injuries and work‐related diseases remain major public health concerns, contributing to preventable mortality, temporary and permanent disability, income loss, and direct and indirect costs for health and social protection systems [1, 2, 3, 4]. In 2019, an estimated 395 million workers experienced non‐fatal occupational injuries or work‐related diseases, and 2.93 million work‐related deaths were recorded, of which 11.3% were attributed to occupational injuries [5, 6].

There was an annual average of 170,429 notifications of work‐related diseases and injuries in Brazil's health system, whereas the Ministry of Social Security recorded an annual average of 651,341 reports in the same period [7]. Within Brazilian epidemiological surveillance, occupational injuries are mandatory notifiable events in the Notifiable Diseases Information System because they are of public health interest. These notifications are universal in scope, as the system operates nationwide and records events across Brazil. In this study, we refer to these as health reports [8, 9].

Despite this, Brazilian studies have documented substantial underreporting across sources and settings. Capture–recapture methods and cross‐system comparisons have estimated underregistration of work‐related deaths and fatal occupational injuries [10]. Other evidence indicates relevant reporting losses, suggesting that observed counts within one system may represent only part of the true burden [11]. One study reported 499,916 notified injuries between 2015 and 2022 and estimated approximately 13,274 underreported injuries during the first year of the pandemic [12].

In addition to health reports, the Ministry of Social Security provides data on occupational injury communications (in Portuguese, Comunicação de Acidente de Trabalho [CAT]). In this study, we refer to these as social security reports. These reports record work‐related diseases and injuries among workers insured under the General Social Security Regime. Their issuance is mandatory for employers, although workers, health services or other institutions may also submit them when employers do not do so [13, 14].

These systems differ in coverage and reporting incentives. Social security reports are strongly conditioned by formal employment and the employer–social security interface, whereas health surveillance within the National Health System has nationwide territorial coverage and includes formal and informal workers. Therefore, municipalities where social security reports exceed health reports may indicate limitations in case detection and recording within surveillance, as well as local operational heterogeneity [14].

Although occupational injury underreporting is well established in Brazil, evidence has mostly come from local studies, specific populations, temporal analyses or individual‐level linkage. These approaches estimate underreporting but provide limited national evidence on spatial discrepancies between systems or priority municipalities for surveillance strengthening. This gap matters because underreporting is unlikely to be spatially random. Accordingly, this study aimed to identify spatial discrepancies between health and social security reports on occupational injuries in Brazil in 2022–2023 and to discuss their implications for workers’ health surveillance.

## 2 Methods

### 2.1 Study Design, Setting and Period

This was a cross‐sectional ecological study with an emphasis on spatial analyses. We used secondary, municipality‐level aggregated data covering all of Brazil, including five macroregions (north, northeast, southeast, south and central‐west), 27 federative units (Figure S1) and 5570 municipality‐level territories. The data refer to occupational injuries occurring between January 2022 and December 2023. This period was selected to minimise potential distortions associated with the COVID‐19 pandemic, particularly changes in labour dynamics and the reporting capacity of information systems. We assumed that, from 2022 onwards, the epidemiological and occupational context in Brazil more consistently reflected post‐pandemic conditions.

### 2.2 Data Sources and Variables of Interest

We used health reports from the Notifiable Diseases Information System (Sinan) from the Ministry of Health, available through the Information Technology Department of the Unified Health System (Datasus). We also used social security reports originating from occupational injury communications forms (CAT) from the Ministry of Social Security, obtained via a request under Brazil's Access to Information Law (protocol #18800.303292/2024‐65). Data were collected in December 2024.

In Brazil, occupational injury records are available through two main national information systems from different institutional sectors. Sinan is the national surveillance system used to record compulsory notifiable diseases and health conditions, including work‐related injuries. Sinan is fed by notifications and investigations conducted by health services and supports workers’ health surveillance within the Unified Health System. Occupational injury communications (CAT), linked to the social security sector, record occupational accidents and work‐related diseases among workers insured under the General Social Security Regime. These social security reports are usually issued by employers, although workers, health services, unions or public authorities may also submit them when employers do not do so. Thus, although Sinan is organised as a health surveillance system and CAT as a social security reporting mechanism, both provide official national data on occupational injuries in Brazil (Table 1) [14].

**TABLE 1:** Information systems characteristics.

Feature	Health reports	Social security reports
Institutional sector	Health surveillance/SUS	Social security/labour‐related reporting
Main purpose	Epidemiological surveillance	Social security and labour communication
Reporting mechanism	Health service notification	Employer, worker, union, health service or other authorised sources
Population theoretically covered	All workers, formal and informal	Mainly formally insured workers
Territorial variables used	General occupational injuries: municipality of occurrence Occupational injuries involving exposure to biological material: municipality of the company	Municipality of employer
Main implication	Broader public health coverage but dependent on recognition and notification by health services	Stronger link to formal employment and benefits but limited for informal work

The territorial variable used differed according to the reporting source and type of occupational injury. For general occupational injury records in Sinan, we used the municipality where the injury occurred. This variable had higher completeness than the municipality of the company (94.1% vs. 81.1%, respectively). In addition, among records with information available for both variables, the municipality of occurrence and municipality of the company were concordant in 91.76% of records. We, therefore, considered the municipality of occurrence the most appropriate territorial variable for general occupational injuries. For occupational injuries involving exposure to biological material, the municipality of occurrence was not available in the corresponding database; therefore, we used the municipality of the company. Similarly, for social security reports, the municipality of occurrence was unavailable, and the municipality of the employer was used. Differences in the territorial variables available across data sources may introduce spatial misclassification when the municipality of the company or employer differs from the municipality where the injury occurred.

### 2.3 Study Population and Exclusion Criteria

The study population comprised all work‐related injury records notified through health reports or social security reports during the study period, including injuries involving exposure to biological material (e.g., injuries occurring in healthcare settings). In this study, occupational injuries were defined as events caused by non‐natural causes occurring in the workplace, on the way to work or during work activities, which may result in injury or functional impairment, with potential disability or death [9].

We excluded records of injuries involving venomous animals (ICD‐10 X20 to X29), exogenous poisonings (ICD‐10 X40 to X49) and events related to interpersonal violence or self‐harm (ICD‐10 X60 to Y20 and Y87). Although these events are external causes and may result in injury, surveillance of these conditions in Brazil does not routinely occur through occupational health surveillance teams and follows condition‐specific reporting protocols, which limits comparability between health reports and social security reports. Finally, we excluded records classified as work‐related diseases (ICD‐10 A00 to Q99 and U04 to U99).

### 2.4 Statistical Analysis

We performed descriptive analyses to quantify the number of occupational injuries captured in health reports and social security reports and applied spatial analytical methods using Brazilian municipalities as the unit of analysis.

We aggregated counts at the municipality level according to the territorial variables defined above and generated total counts for both health reports and social security reports for 2022 and 2023. To quantify discrepancies between systems, we calculated a municipality‐specific ratio of health reports to social security reports, where values below one indicate relatively fewer health reports than social security reports. We used absolute counts because comparable municipality‐level denominators were not available for both systems, particularly given the broader theoretical population covered by health reports and the formal employment restriction inherent to social security reports. Therefore, the analysis focused on spatial discrepancies in reported counts rather than rate‐based differences.

Because a few municipalities had zero counts in one of the systems when the other had at least one, we implemented a prespecified rule for missingness and zero handling. Municipalities with both health reports and social security reports equal to zero were coded as missing, because the ratio is undefined and uninformative when neither system recorded any events. For all other municipalities, we calculated a municipality‐specific log‐ratio as

$$LR_{i}=\;\ln\left(\frac{S_{i}+0.5}{C_{i}+0.5}\right)$$

where LR*_i_* is the log‐ratio for municipality i, $S_{i}$ is the health reports count in municipality i, and $\;C_{i}$ is the social security reports count in municipality i.

This transformation yields a symmetric metric centred at zero, where positive values indicate relatively more health reports than social security reports, negative values indicate relatively more social security reports than health reports, and values near zero indicate similar counts. The 0.5 continuity correction was applied to avoid undefined ratios in municipalities with zero counts in one system, although we recognise that this choice may have greater influence where the number of reported events is very small. The log‐ratio variable was exported with the municipal geospatial layer and analysed in GeoDa to quantify global spatial autocorrelation and identify local clusters of discrepancy using local indicators of spatial association.

The health reports/social security reports log‐ratio should be interpreted as an indirect indicator of discrepancy between reporting systems. Negative values, where social security reports exceed health reports, do not directly measure the true magnitude of underreporting. However, because Sinan is intended to support universal occupational health surveillance, these values may indicate territories where events already recognised through the social security reporting pathway may not be adequately captured by health surveillance.

### 2.5 Moran Analysis and Spatial Clustering

We calculated the global Moran's *I* for municipality‐level counts of health reports, social security reports and the health reports/social security reports log‐ratio to assess overall spatial autocorrelation across municipalities [15]. We also calculated the local Moran's *I* to map statistically significant clusters. This analysis classifies municipalities into four cluster types based on the combination of values in a municipality and its neighbours: high–high, low–low, low–high and high–low [15]. We constructed a first‐order queen contiguity spatial weights matrix, in which neighbouring municipalities share at least one boundary point, and used 999 permutations with a statistical significance threshold of *p* < 0.05.

Given the aim of identifying areas of potential underreporting in health reports, we interpreted municipalities in which social security reports exceed health reports as areas potentially consistent with underreporting in the health surveillance system, because health surveillance is expected to capture occupational injuries among formal and informal workers, whereas social security reports pertain to insured formal workers. This interpretation should be understood as an indirect indicator of discrepancy between systems rather than a direct measure of underreporting.

All data management was conducted in R and RStudio (R version 4.5.1) [16]. Graphs were produced in Microsoft Excel (version 2016). Spatial analyses were performed in GeoDa (version 1.22), and maps were prepared in QGIS (version 3.30.1).

### 2.6 Sensitivity Analyses

Two sensitivity analyses were conducted to assess the robustness of the spatial findings. First, to reduce the influence of unstable ratios in municipalities with small numbers of reports, municipalities with fewer than 10 notifications in either system were excluded from this sensitivity calculation. In this restricted dataset, the Sinan/CAT log‐ratio was recalculated without the 0.5 continuity correction, using only municipalities with at least 10 Sinan reports and at least 10 CAT reports (Supporting Information).

Second, we repeated the analysis using report rates instead of absolute counts. Population denominators were obtained from the 2022 Brazilian Census, accessed through the SIDRA platform, table 10,262 [17]. For Sinan, rates were calculated per 100,000 occupied workers, using the total occupied population as the denominator. For CAT, rates were calculated per 100,000 formally employed workers, reflecting the formal employment coverage of the social security reporting system. We then calculated a rate‐based log‐ratio comparing Sinan report rates with CAT report rates. Spatial autocorrelation and local cluster analyses were repeated using the same spatial weights and statistical procedures applied in the main analysis (Supporting Information).

## 3 Results

During the study period, we analysed 745,840 occupational injury records from health reports (2022: 312,677; 2023: 433,163) and 949,466 occupational injury records from social security reports (2022: 411,255; 2023: 538,211). Among Brazil's 5570 municipalities, health reports covered 5466 (98.1%), whereas social security reports covered 4509 (80.9%). Regionally, the southeast showed the largest discrepancy, with more social security reports (516,069) than health reports (300,291). In the north, health reports totalled 42,876 and social security reports 35,407; in the northeast, health reports totalled 94,605 and social security reports 93,465; in the south, health reports totalled 236,780 and social security reports 230,796; and in the central‐west, health reports totalled 71,288 and social security reports 73,729.

Figure 1 shows that most of the Brazilian territory had occupational injury records in both systems (*n* = 4470). Municipalities with health reports only were concentrated in the north and northwest, whereas municipalities with social security reports only were more dispersed and occurred predominantly in the southeast and south. In addition, 66 (1.0%) municipalities had no records in either database and were, therefore, not included in the comparative spatial analysis (Figure 1).

**FIGURE 1:**
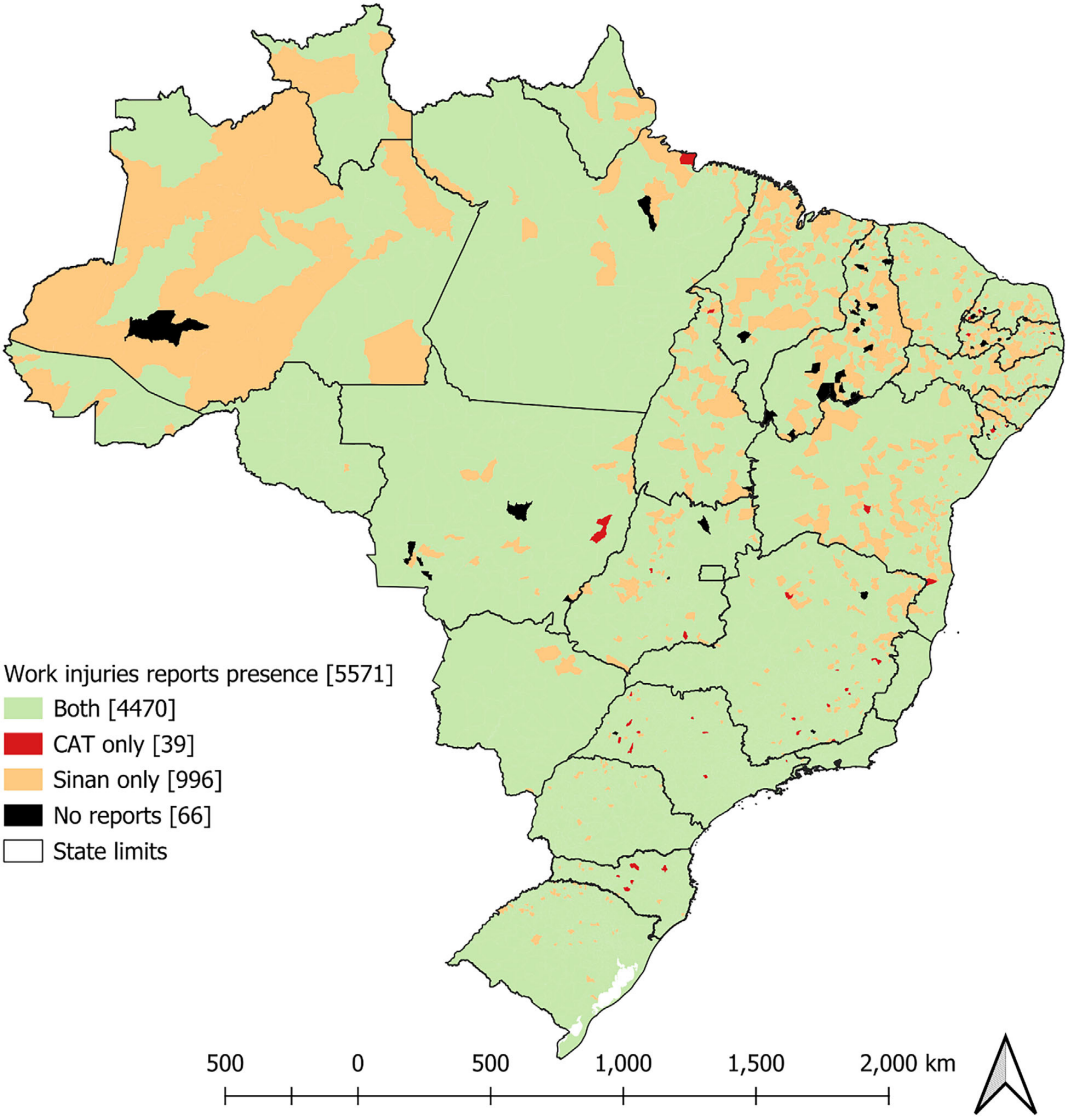
Spatial distribution of the presence of health reports and social security reports in Brazilian municipalities, 2022–2023.

Regarding the spatial distribution of occupational injuries, Figure 2A shows that most municipalities in the country recorded fewer than 500 occupational injuries in health reports. Municipalities reporting 500 or more occupational injuries were concentrated in the south, as well as in Campo Grande (central‐west) and São Paulo (southeast) (Figure 2A).

**FIGURE 2:**
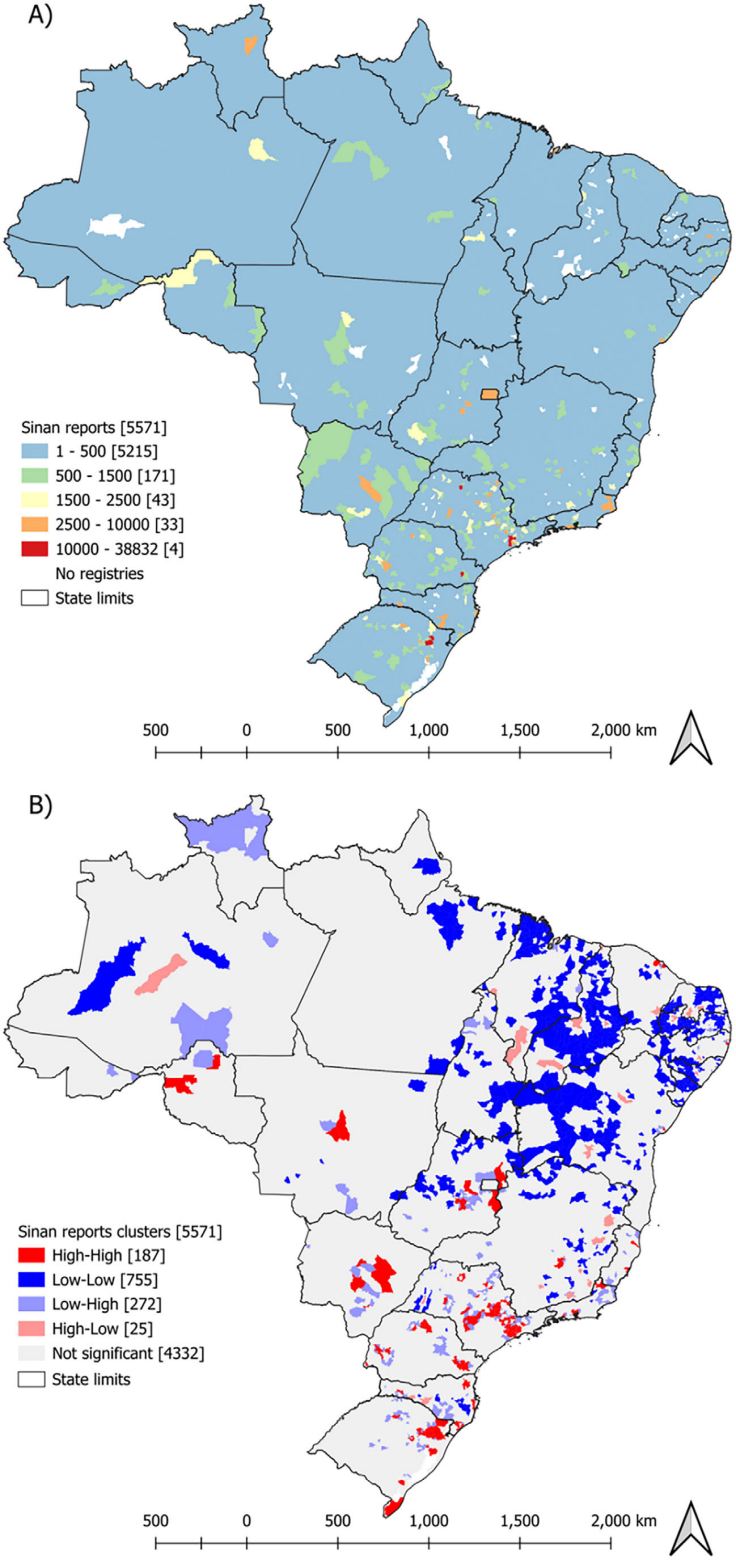
Distribution of health reports in Brazilian municipalities, 2022–2023. (A) Spatial descriptive distribution of health reports; (B) spatial clusters of health reports.

Global spatial autocorrelation was positive but weak for health reports (Moran's *I* = 0.101; Figure S2). In the local spatial analysis, most municipalities were classified as not significant for health reports (*n* = 4332, 77.8%). Local cluster analysis results showed 187 (3.4%) municipalities in high–high clusters, 755 (13.5%) municipalities in low–low clusters, 272 (4.9%) municipalities in low–high clusters and 25 (0.4%) municipalities in high–low clusters. high–high clusters of health reports were more frequent in municipalities located in Rio Grande do Sul (south) and São Paulo (southeast). In contrast, low–low clusters of health reports were concentrated in the northern part of the country, with a large concentration in Minas Gerais (southeast) and widespread low–low clustering across the northeast (Figure 2B).

A broadly similar pattern was observed for social security reports. Figure 3A shows that most municipalities in the northern part of Brazil recorded fewer than 500 occupational injuries in social security reports, whereas higher counts were concentrated in the southern part of the country, particularly in the state of São Paulo (Figure 3A).

**FIGURE 3:**
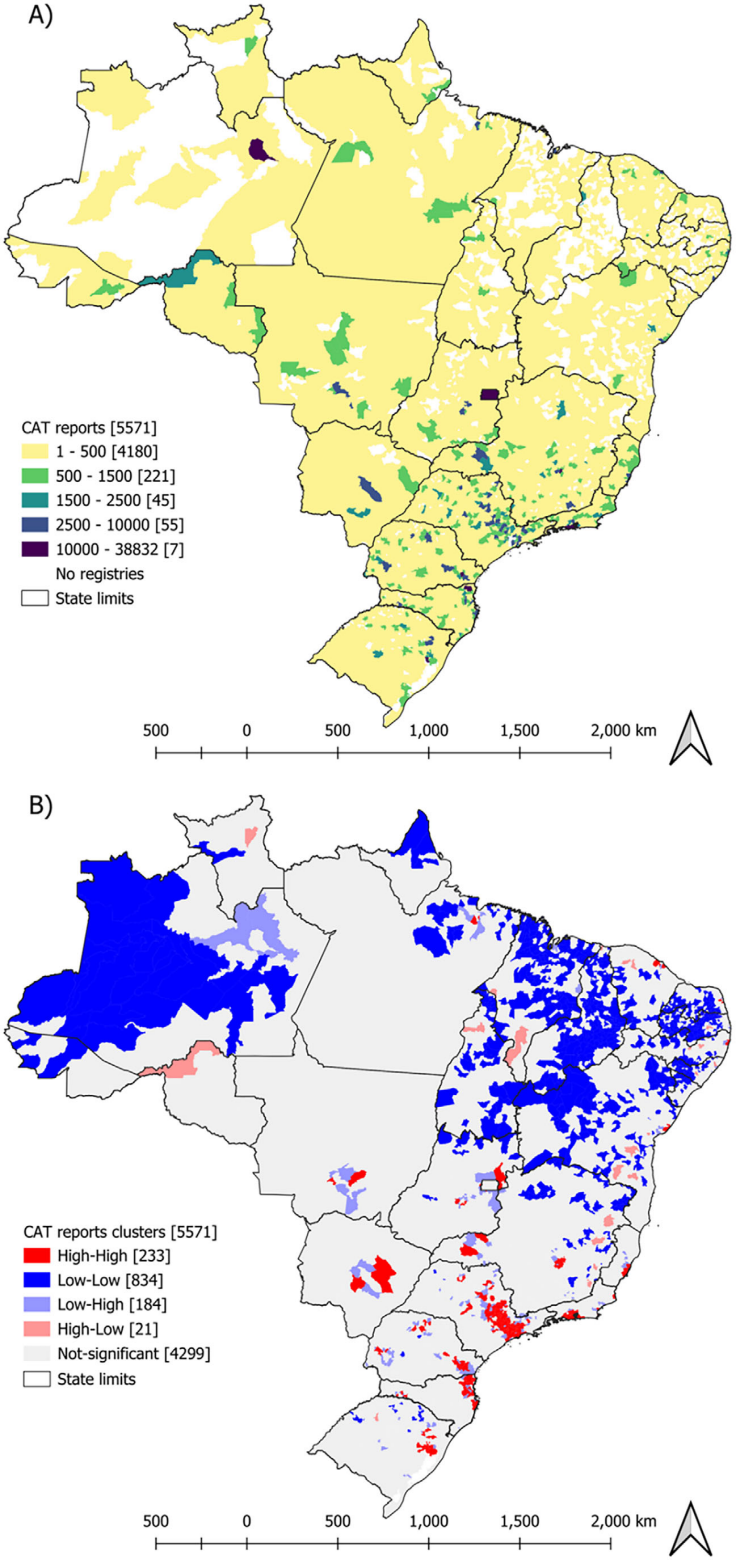
Distribution of social security reports in Brazilian municipalities, 2022–2023. (A) Spatial descriptive distribution of social security reports; (B) spatial clusters of social security reports.

Global spatial autocorrelation was positive but weak for social security reports (Moran's *I* = 0.131; Figure S3). In the local spatial analysis, most municipalities were classified as not‐significant for social security reports (*n* = 4299, 77.1%). LISA results showed a similar pattern to the health reports clusters, with 233 (4.2%) municipalities in high–high clusters, 834 (15.0%) municipalities in low–low clusters, 184 (3.3%) municipalities in low–high clusters and 21 (0.4%) municipalities in high–low clusters. Low–low clusters of social security reports were widespread in the state of Amazonas (north) and across the northeast, indicating spatial clustering of municipalities with low occurrence of social security reports. High–high clusters, indicating spatial clustering of municipalities with high occurrence of social security reports, were more frequent around state capitals in the south, in Campo Grande (central‐west) and in São Paulo and Rio de Janeiro (southeast) (Figure 3B).

Finally, Figure 4A shows the spatial distribution of the log‐ratio between health reports and social security reports. Although Figure 1 indicates a low number of municipalities with social security reports only, Figure 4A shows a high number of municipalities where social security reports exceeded health reports (*n* = 1418; 25.4%), mainly in the southeast, central‐west and Santa Catarina state. These municipalities (social security reports > health reports) were frequently located near large urban centres, particularly around state capitals, with the exception of some capitals in the north (Figure S4). Meanwhile, municipalities where health reports exceeded social security reports (*n* = 3998; 71.8%) were concentrated in the north and northeast and also in Rio Grande do Sul and Curitiba (south). A total of 89 (1.5%) municipalities had identical counts in both systems.

**FIGURE 4:**
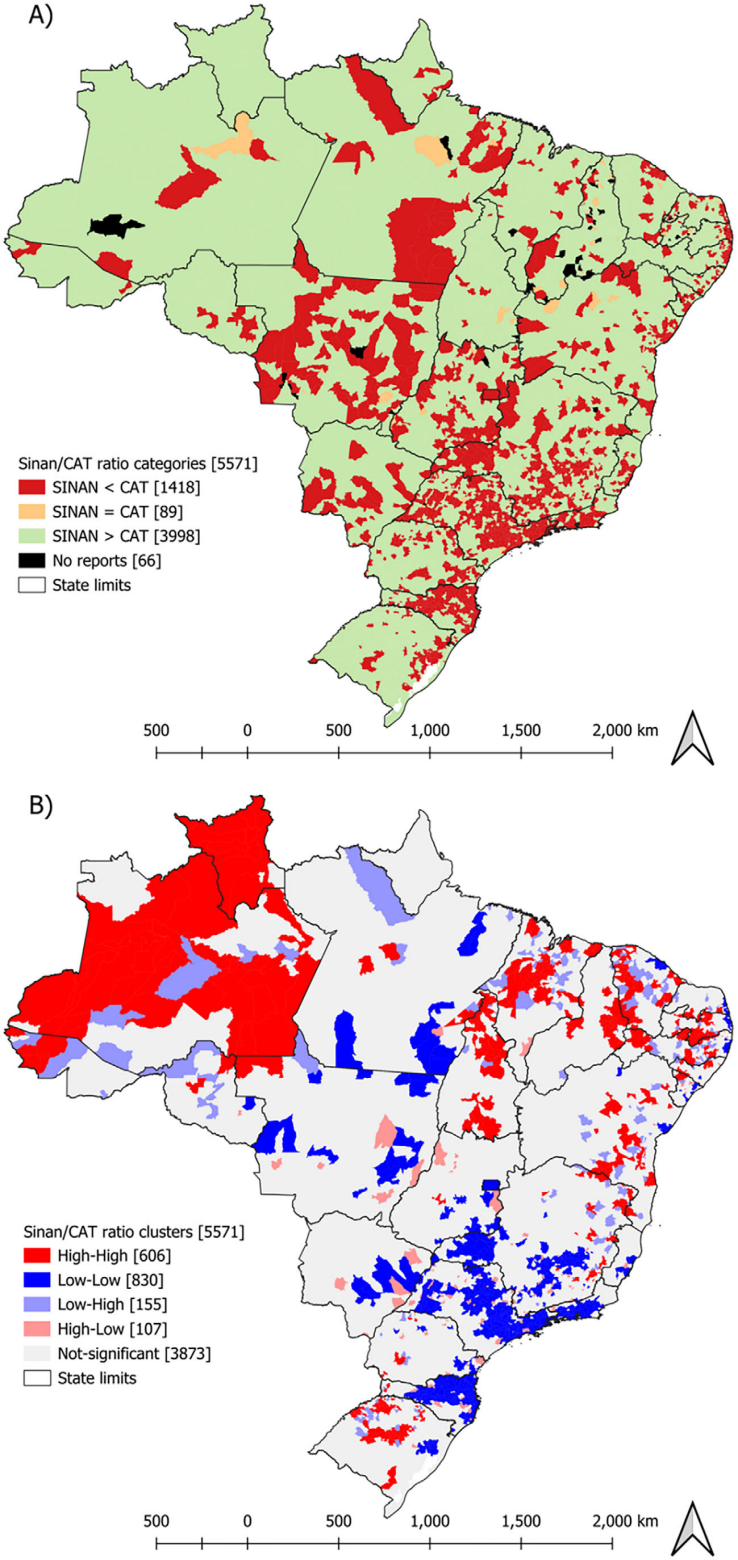
Spatial distribution of the ratio between health reports and social security reports in Brazilian municipalities, 2022–2023. (A) Distribution of system report proportions; (B) spatial clusters of the log‐ratio.

Global spatial autocorrelation for the health reports/social security reports ratio was stronger (Moran's *I* = 0.391; Figure S5), indicating a more structured geographic pattern in the discrepancy between the two reporting systems. In the local spatial analysis, fewer municipalities were classified as not‐significant for the health reports/social security reports ratio (*n* = 3873, 69.5%) than for either system separately. For the health reports/social security reports log‐ratio, Figure 4B shows 606 (10.9%) municipalities in high–high clusters, 830 (14.9%) municipalities in low–low clusters, 155 (2.8%) municipalities in low–high clusters and 107 (1.9%) municipalities in high–low clusters. Low–low clusters of the log‐ratio indicate municipalities with lower values clustered with neighbouring municipalities with similarly low values, corresponding to areas where social security reports consistently exceeded health reports. In contrast, high–high clusters indicate areas where health reports consistently exceeded social security reports. High–high clusters were more frequent in the north, particularly in Amazonas, Roraima and Tocantins, and in parts of the northeast. Low–low clusters were more frequent in the central‐west and southeast and in the state of Paraná (south). With the exception of the north, areas surrounding most state capitals were predominantly classified as low–low clusters, including in the northeast (Figure 4B).

In sensitivity analysis, which included only municipalities with at least 10 health reports and at least 10 social security reports, the number of high–high clusters where health reports exceeded social security reports decreased considerably in the north, particularly in Amazonas and Roraima and across most of the northeast. Conversely, the number of high–high clusters where health reports exceeded social security reports increased in the south, including areas that were not statistically significant in the main analysis. Consistent with the main analysis, low–low clusters, corresponding to areas where health reports were less frequent than social security reports, remained in the southeast, particularly in São Paulo and Rio de Janeiro (Figure S6).

In sensitivity Analysis B, using population‐based rates, the spatial pattern changed, with a reduction in the size of high–high clusters where health reports exceeded social security reports. However, these clusters persisted in Roraima and in the southern portion of Amazonas. High–high clusters also decreased in the northeast but increased in the south. Low–low clusters, corresponding to areas where social security report rates exceeded health report rates, decreased in Mato Grosso do Sul, although their distribution remained similar in parts of the north, northeast, southeast and south. Some municipalities previously classified as low–low were reclassified as high–low, indicating municipalities with higher health report rates surrounded by neighbours with lower health report rates (Figure S7).

## 4 Discussion

In this study, most Brazilian municipalities reported occupational injuries in at least one system. Areas with higher social vulnerability, particularly in the north and northeast, showed lower coverage of social security reports and higher occurrence of occupational injuries in health reports. However, the total number of social security reports exceeded health reports, although Ministry of Health notification instruments are intended to cover both formal and informal workers [14].

Sinan and CAT capture events through different institutional pathways. Because Sinan supports universal health surveillance across all workers, whereas CAT is mainly linked to formally insured employment, areas where social security reports exceed health reports may indicate events already recognised through the social security pathway but not adequately captured by health surveillance. These areas should not be interpreted as direct measures of underreporting but as operational markers of potential underreporting in workers’ health surveillance.

These findings are compatible with potential underreporting in the health system. Multiple factors may contribute to this gap, including limited healthcare seeking, fragmented communication between social security and health services, difficulties in recognising the relationship between the event and work and notification failures due to negligence, limited technical confidence or lack of knowledge. Workers, particularly those in precarious employment, may also fear retaliation when reporting occupational injuries [14, 18, 19, 20]. These explanations should be interpreted as plausible mechanisms supported by the literature, rather than drivers directly tested in this analysis.

Municipalities in the south and southeast had a high occurrence of occupational injuries in both systems. This pattern aligns with the economic profile of these regions, which concentrate more complex and formal productive processes, more workers insured by social security, greater employer oversight, larger companies and greater industrial activity [21]. In the north and northeast, lower recorded occurrence is consistent with lower population density and economic activity that is, on average, less industrialised than in large urban centres [21, 22].

The spatial approach used in this study is consistent with previous research on occupational injuries that applied spatial autocorrelation methods, including Moran's *I*, to identify geographic patterns of severe occupational injuries in the south‐eastern United States. Although our study focuses on discrepancies between reporting systems rather than injury risk itself, both approaches use spatial methods to identify geographically structured patterns [23]. This provides methodological support for the use of Moran's *I* in the present study to identify territorial concentrations of reporting discrepancies and areas that may warrant targeted surveillance actions.

This study also showed more clusters in which health reports exceeded social security reports in the north and northeast, where informal employment is substantial. In 2022, 42.1% of all jobs in Brazil were informal, with higher informality in these regions [5, 22]. Workers’ health surveillance aims to promote health, prevent morbidity and mortality, and reduce work‐related risks and vulnerabilities [9]. In Brazil, this work is mainly implemented through Workers’ Health Reference Centres (Cerest), which support surveillance actions and the wider healthcare network [9].

The contrast between the north and northeast and the southeast and south, where clusters consistent with potential underreporting in health reports were concentrated, highlights operational challenges for occupational injury notification. Although reporting among formally employed workers could be more straightforward, these workers may use private health services more frequently [24]. Private services may not notify consistently, even when reporting is mandatory [25]. In addition, the operational capacity of workers’ health surveillance varies across municipalities. Not all territories have local technical references and in many settings Cerest may be the only structure conducting workers’ health surveillance, often with limited epidemiological capacity [14, 26, 27].

Underreporting may occur in both systems. Injured workers may neither seek healthcare and be recorded in health reports nor report to social security and generate social security reports [11]. This may reflect limited awareness of available care and workplace pressures that discourage reporting, as employers may be monitored based on the number of social security reports issued [28]. More broadly, workers’ health policies are not equally effective across the national territory. Their effectiveness depends on political commitment, continuity of action across government levels and sustained surveillance capacity [14].

Strengthening national workers’ health surveillance and supporting training of health services to improve recognition of the relationship between the event and work and strengthen recording in health information systems would improve understanding of occupational injuries and work‐related illnesses and reduce underreporting. This would support the National Policy on Workers’ Health, which emphasises the protection and promotion of workers’ health [29], and contribute to progress towards the Sustainable Development Goal on decent work [30].

### 4.1 Strengths and Limitations

This study provides a national municipal‐level spatial assessment of discrepancies between health surveillance and social security reports of occupational injuries in Brazil. By identifying spatial clusters where social security reports exceed health reports, the analysis may support geographically targeted investigation, training and strengthening of epidemiological and workers’ health surveillance actions. The approach used here can also be applied to other health conditions, whether work‐related or not, to investigate underreporting where different information systems overlap or complement each other.

The reduction of high–high clusters in the north and northeast in sensitivity Analysis A suggests that some areas where health reports exceeded social security reports were influenced by municipalities with low numbers of reports. This may be explained by the fact that health reports include informal workers. Therefore, in areas with low recorded occurrence of occupational injuries, the few events detected may be more frequently captured by health surveillance than by social security reports.

Limitations include the absence of nominal data, which prevented record linkage between health and social security reports, restricting the analysis to aggregated municipality‐level discrepancies and making it impossible to verify whether a CAT report corresponds to a case missing from Sinan. The use of different territorial variables is another key limitation: Health reports primarily used municipality of occurrence, whereas social security reports used municipality of employer. This may create apparent municipality‐level discrepancies, especially in metropolitan areas with commuting. The cluster‐based strategy partly mitigates this limitation by focusing on groups of neighbouring municipalities, but it does not remove the possibility of spatial misclassification. Although sensitivity analyses using rates and excluding small‐count municipalities were performed, residual sensitivity to denominator choice and sparse data may remain. Local Moran results may also be affected by the choice of first‐order queen contiguity weights and by multiple local significance tests. Queen contiguity was considered appropriate because intermunicipal interactions in Brazil often extend across shared borders or vertices and may reflect shared geographical characteristics, health regions and state‐level health system organisation.

Additionally, differences in data collection standards between the two systems may affect comparability. Finally, we did not analyse work‐related diseases; therefore, no inference should be made for these outcomes.

## 5 Conclusions

Spatial patterns suggestive of potential underreporting of occupational injuries in health reports were identified mainly in the southeast and around state capitals, except in the north. Clusters with higher occurrence of occupational injuries in health reports were more frequent in the north and northeast. These findings indicate priority areas for workers’ health surveillance strengthening, including local investigation, improved notification practices and better integration between health and social security information systems.

## Author Contributions

Luiza Maria Parise Morales was the lead investigator of this study and wrote the first draft. Data analysis and validation were performed by Luiza Maria Parise Morales and Klauss K. S. Garcia. Samara Carolina Rodrigues co‐supervised the study, and Klauss K. S. Garcia was the main supervisor. All authors contributed to the conception or design of the study, participated in the investigation and interpretation of the findings, revised the final version, approved the final manuscript and agreed to be accountable for all aspects of the work.

## Funding

The authors have nothing to report.

## Ethics Statement

This study used anonymised, aggregated data and therefore did not require ethics committee approval, in accordance with Brazilian National Health Council Resolution No. 510, dated 7 April 2016. All health report data were accessed through public portals, and social security reports were obtained through an official request via the Access to Information platform (#18800.303292/2024‐65). Data were accessed on 10 December 2024, and authors had no access to information that could identify individual participants during or after data collection.

## Consent

Patient consent was not required because this study used anonymised, aggregated secondary data without individual identifiers.

## Conflicts of Interest

The authors declare no conflicts of interest.

## Supporting information




**Supporting File 1**: puh270400‐sup‐0001‐SuppMat.csv

**Supporting File**: puh270400‐sup‐0002‐SuppMat.csv

**Supporting File** puh270400‐sup‐0003‐SuppMat.csv

**Supporting File**: puh270400‐sup‐0004‐SuppMat

**Supporting File**: puh270400‐sup‐0005‐SuppMat.pdf

**Supporting File**: puh270400‐sup‐0006‐SuppMat.csv

**Supporting File**: puh270400‐sup‐0007‐SuppMat.csv

**Supporting File**: puh270400‐sup‐0008‐SuppMat

## Data Availability

All data used in this study are available as Supporting Information in CSV format, together with the R script used for data analysis.
