# Supplementary material for: Spatial Discrepancies Between Health and Social Security Reports as Markers of Potential Underreporting of Occupational Injuries in Brazil, 2022–2023
Source: Public Health Chall. 2026 Sep 30;5(4):e70400. doi: 10.1002/puh2.70400 (PMC13627980; doi:10.1002/puh2.70400)

SUPPLEMENTARY FILES

Supplementary Figure 1. Study site regions, Brazil.

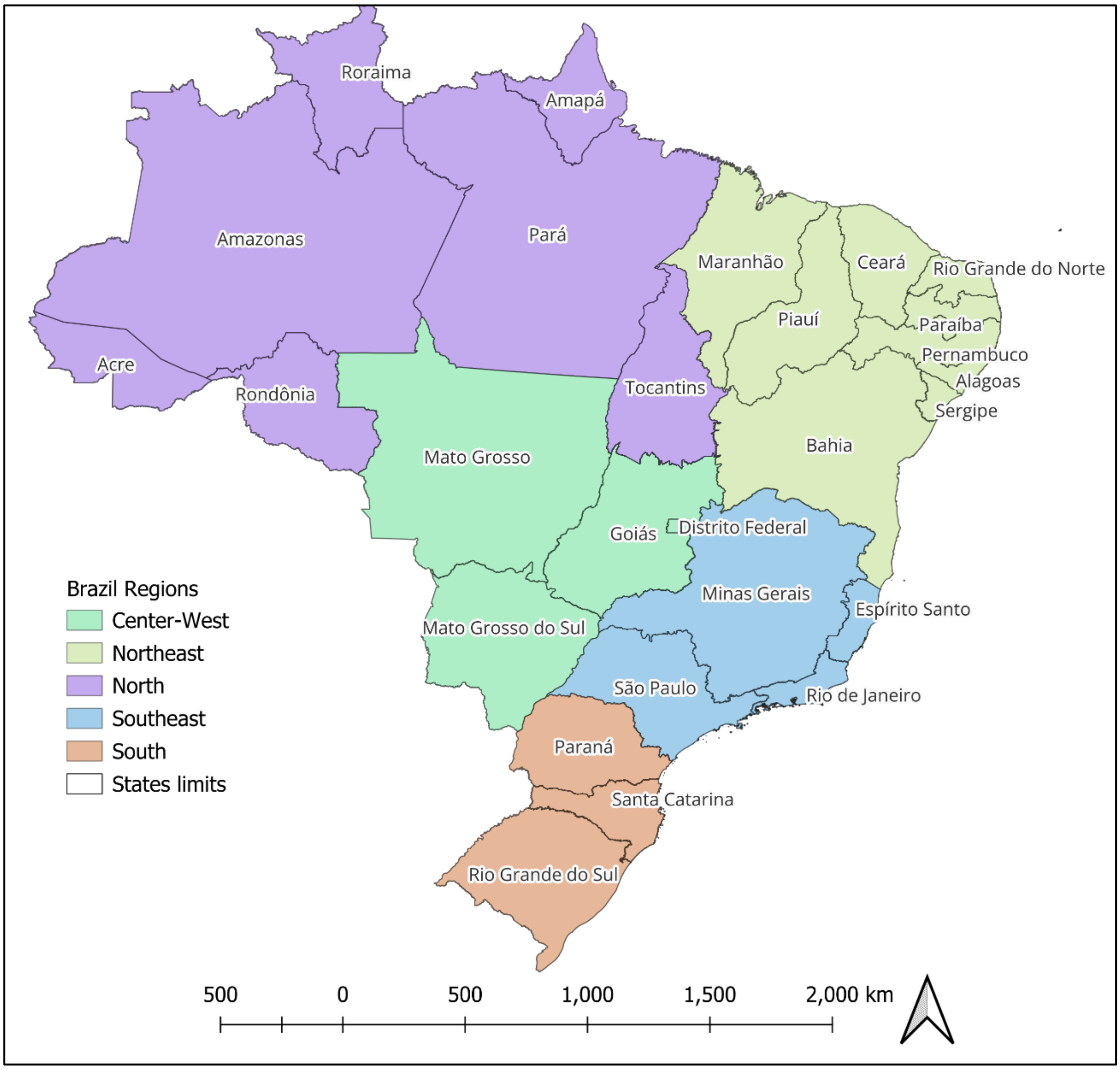

Supplementary Figure 2. Spatial autocorrelation of Sinan reports across Brazilian municipalities: Moran's I scatterplot and LISA cluster map.

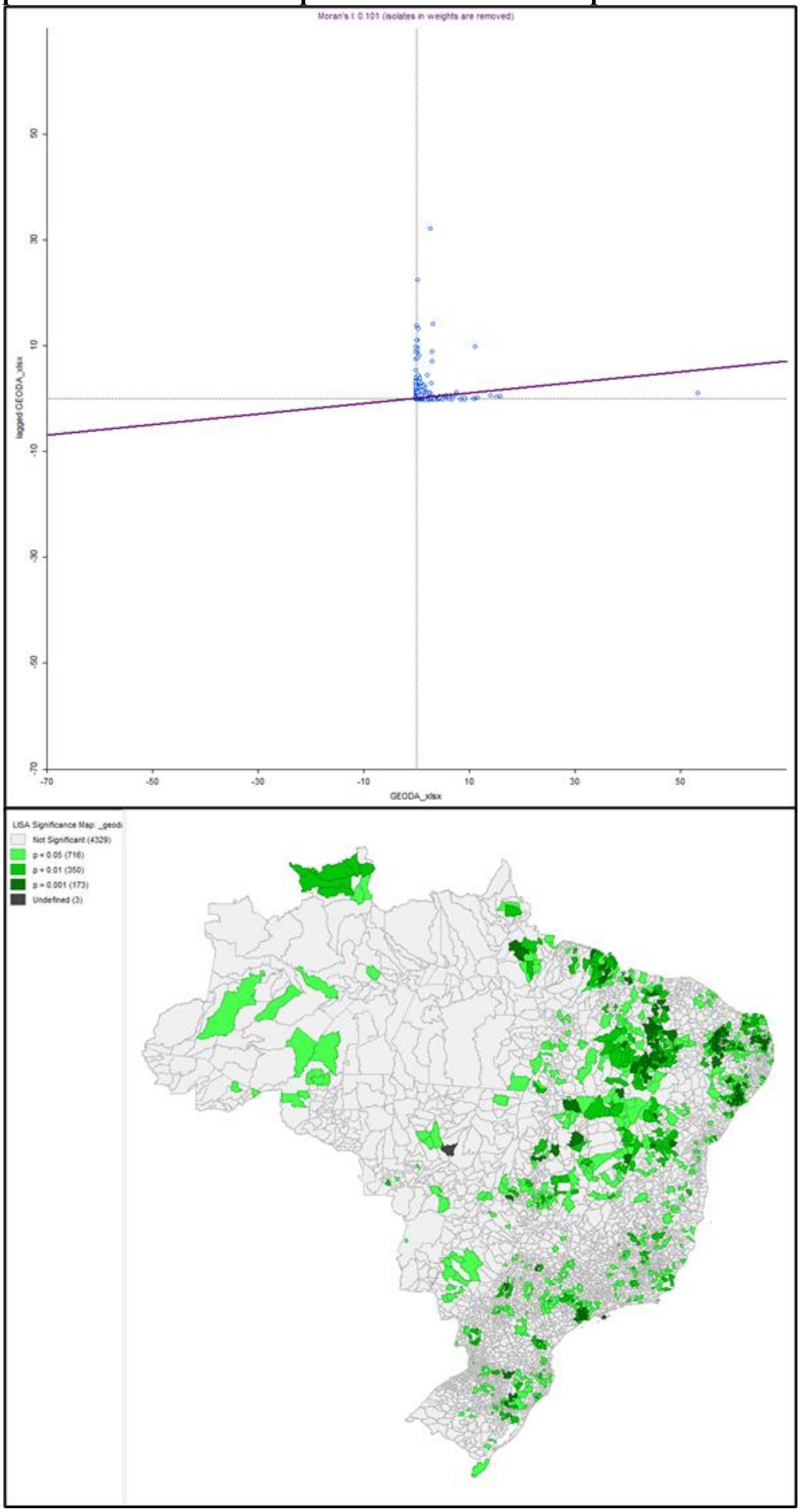

Supplementary Figure 3. Spatial autocorrelation of CAT reports across Brazilian municipalities: Moran’s I scatterplot and LISA cluster map.

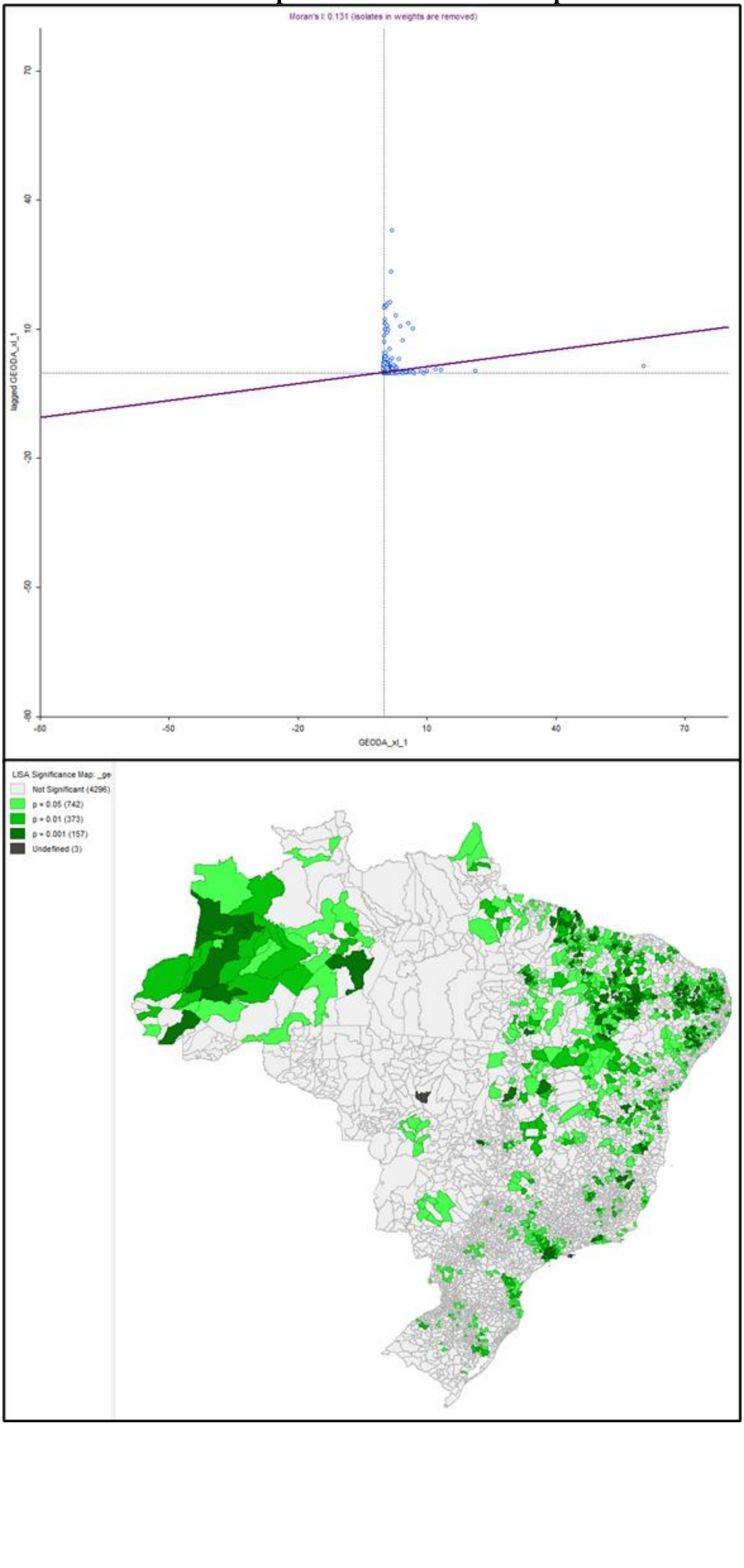

Supplementary Figure 4. Distribution of CAT reports in Brazilian municipalities with capitals represented, 2022 to 2023.

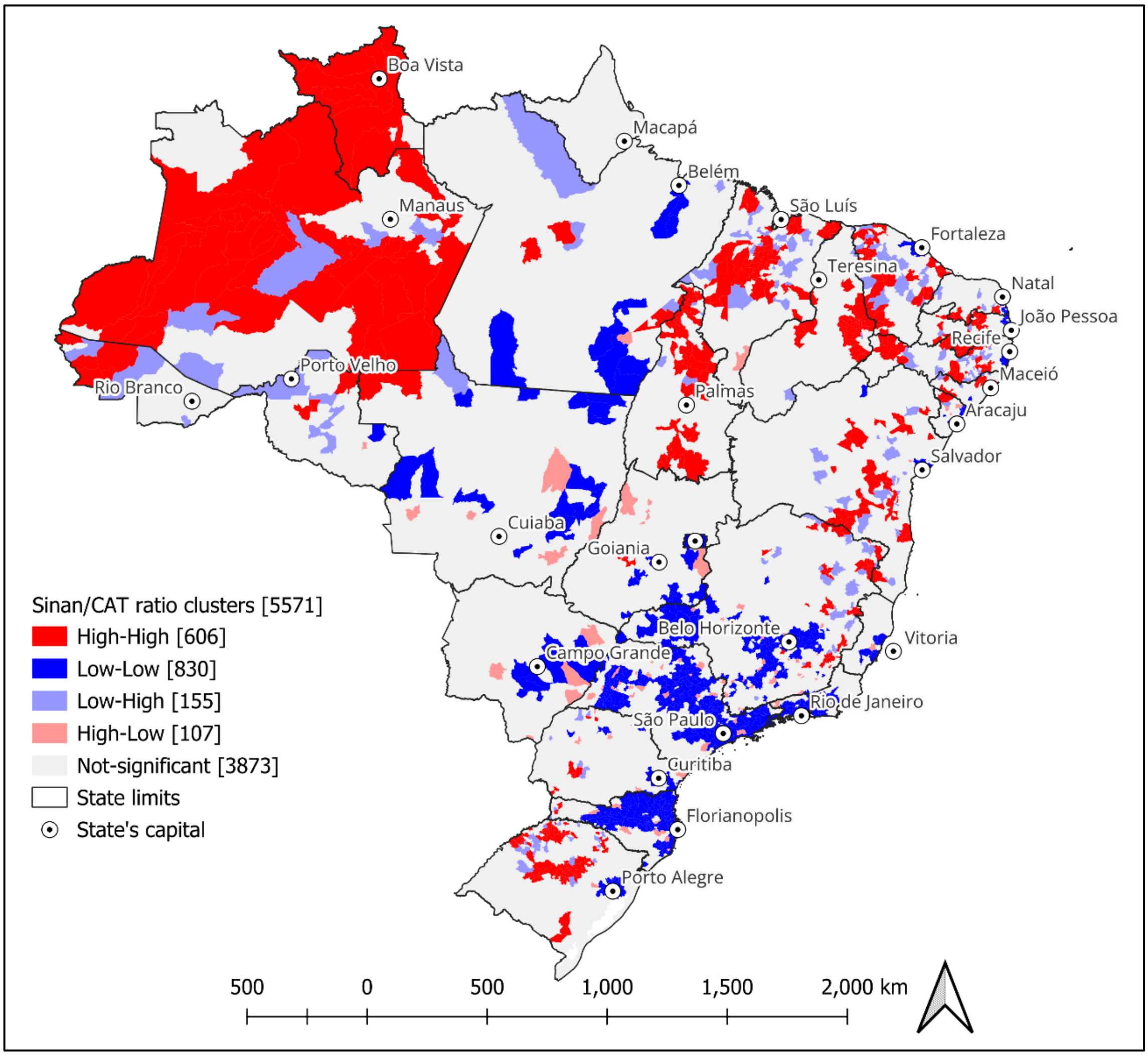

Supplementary Figure 5. Spatial autocorrelation of the Sinan/CAT ratio across Brazilian municipalities: Moran's I scatterplot and LISA cluster map.

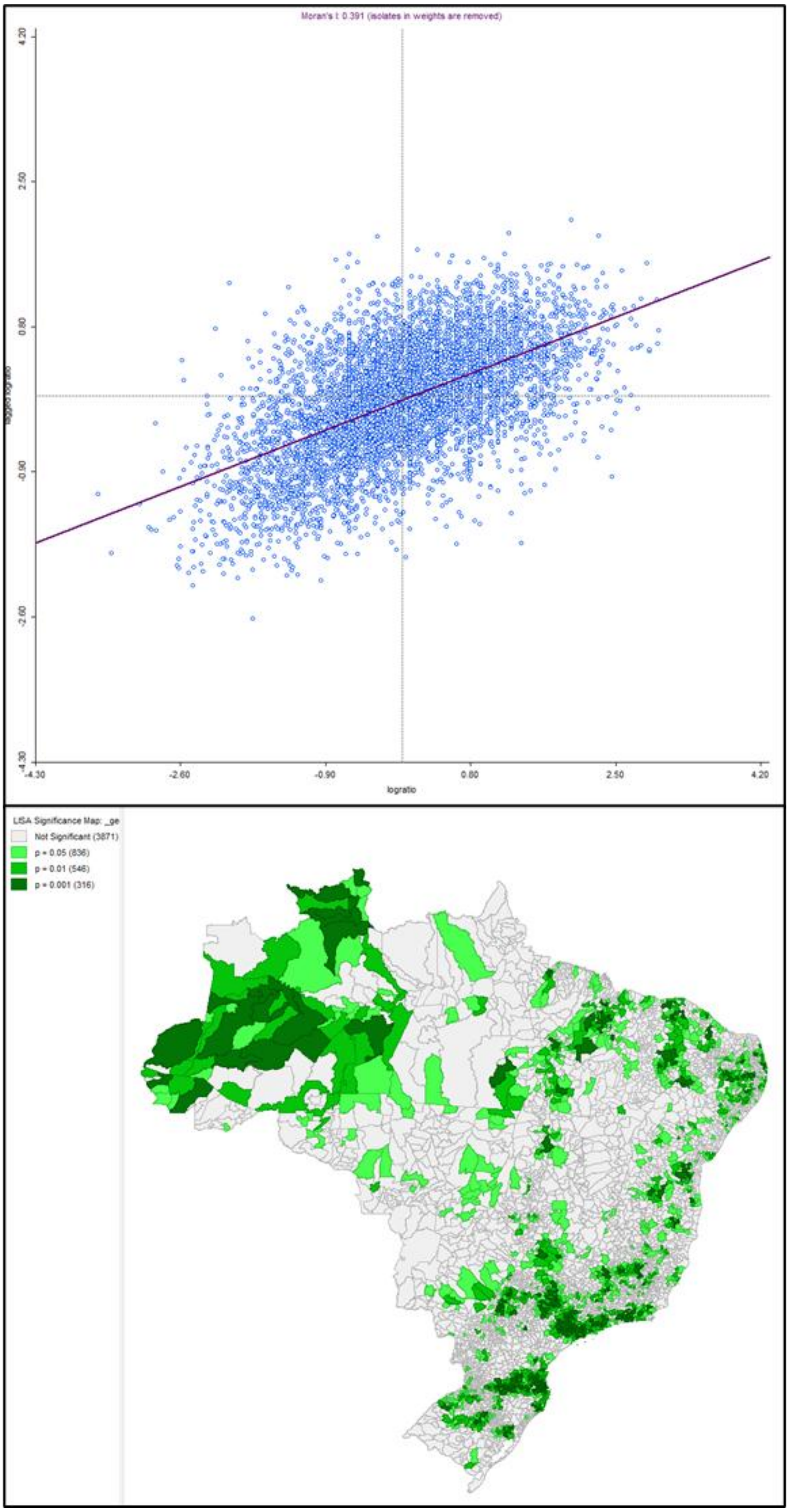

Supplementary Figure 6. log-ratio without municipalities with less than 10 reports.

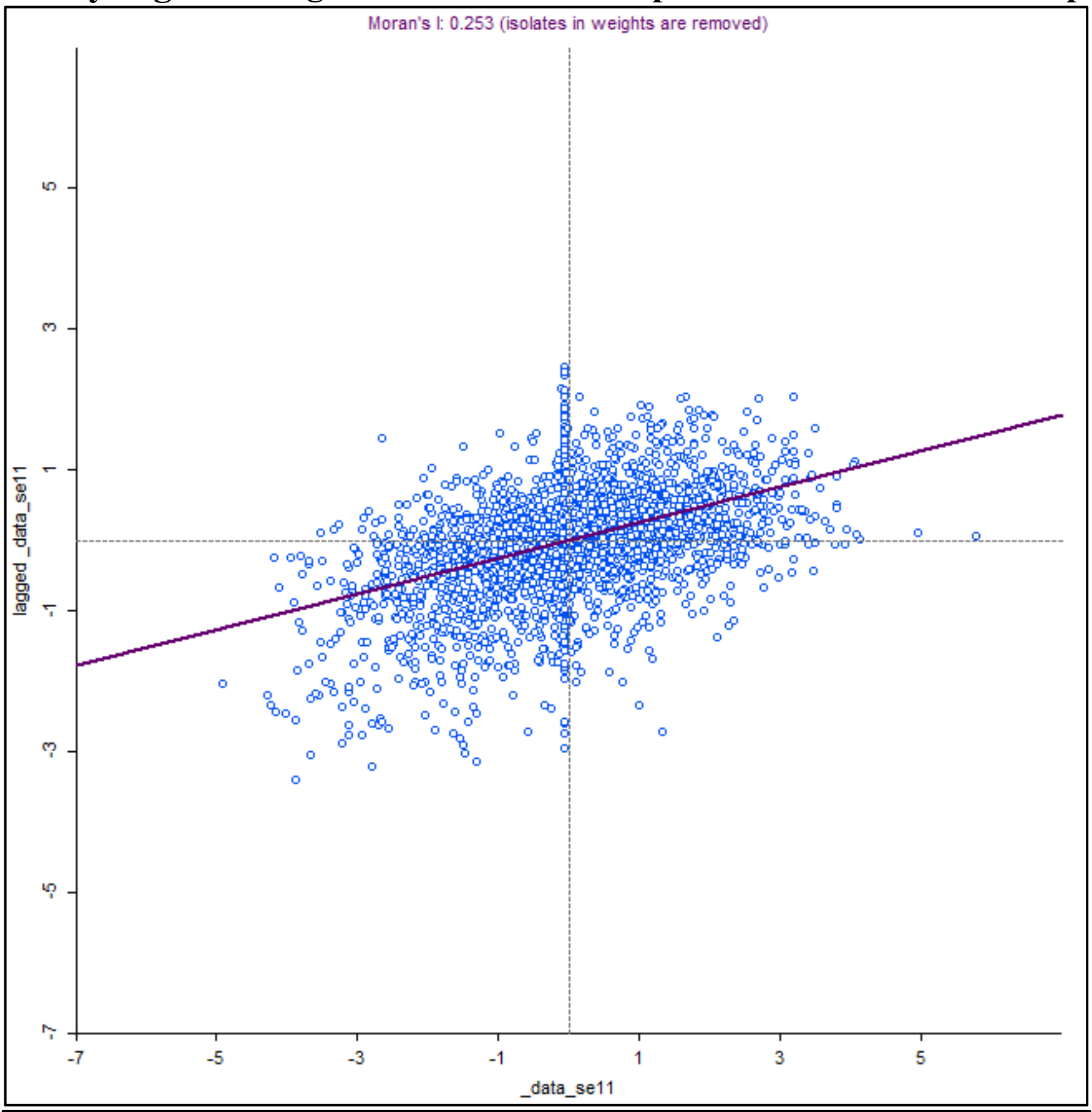

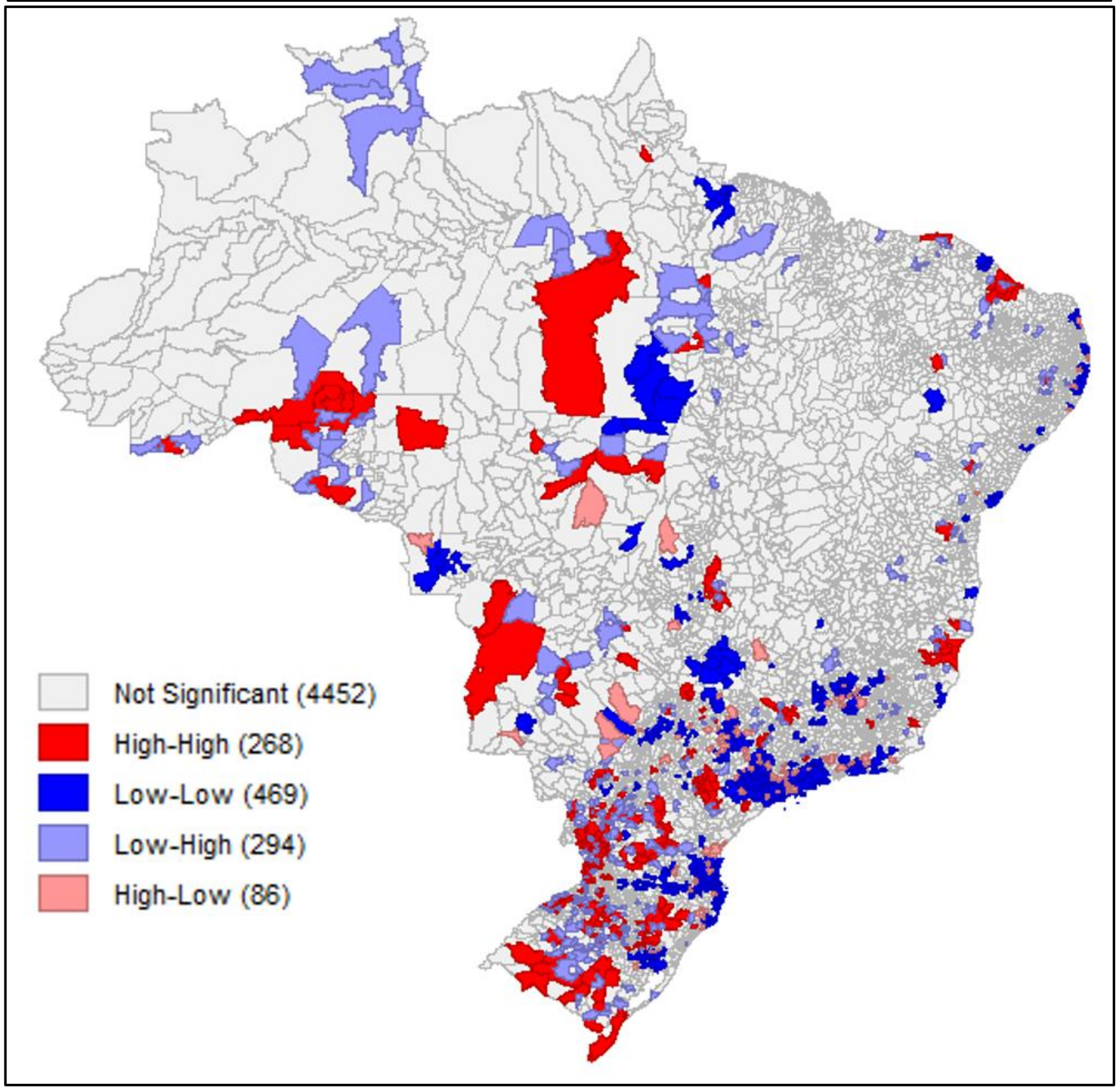

Supplementary Figure 7. log-ratio of Sinan reports rate and CAT reports rate.

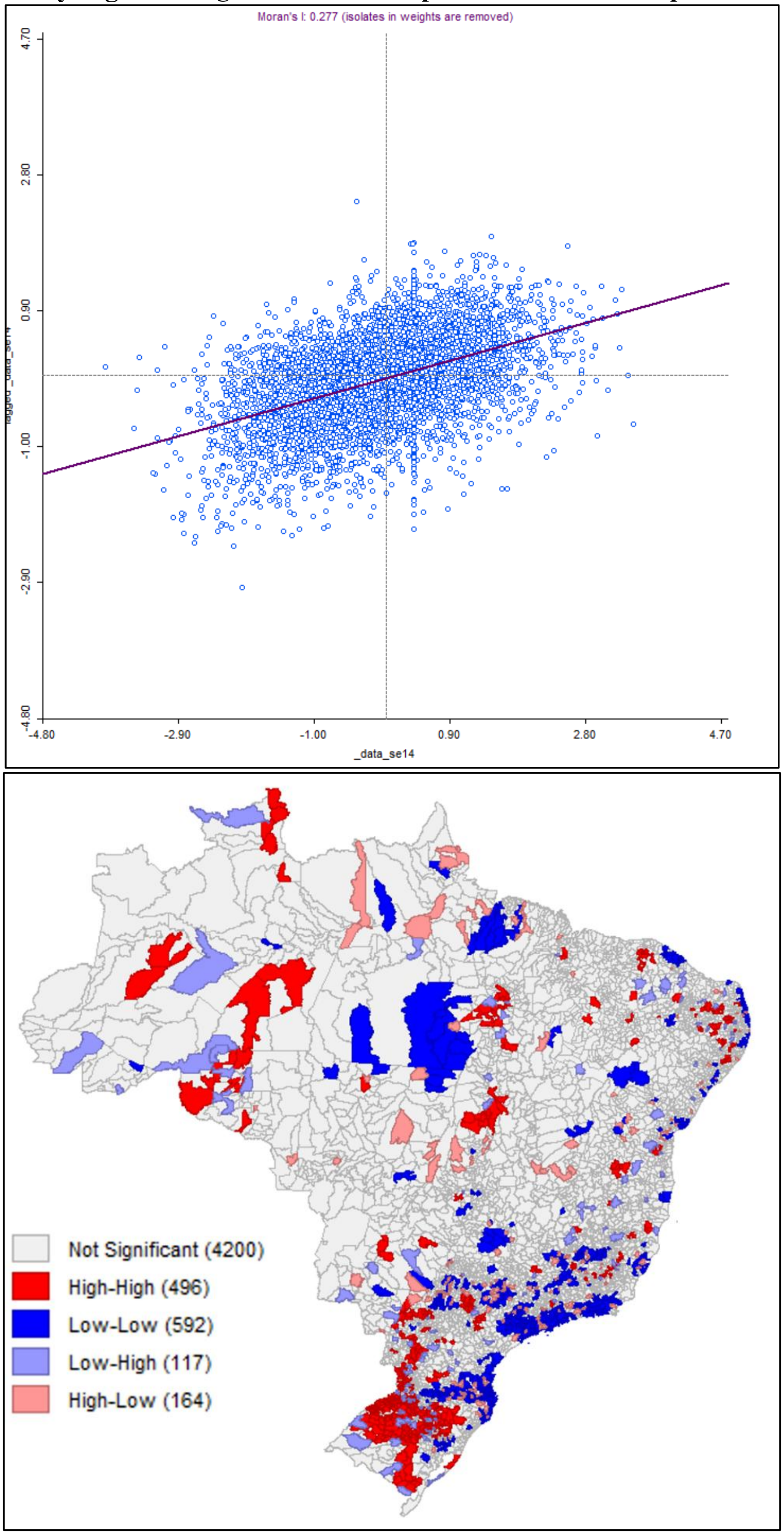

Supplement: Supplementary file 1 [file PUH2-5-e70400-s006.pdf]
